# Medical and Physical Intelligence

**Published:** 1801-01

**Authors:** 


### MEDICAL AND PHYSICAL INTELLIGENCE.

### [ FOREIGN AND DOMESTIC. ]

### *Report of the Committee on the Vaccine Inoculation at Paris.*

SINCE the 3d of Thermidor, the committee have communicated nothing to the public with regard to theſe inoculations. This long interval has not been loſt; the committee are of opinion that they have employed it to advantage.

The firſt experiments, as is generally known, were made with vaccine matter which had been ſent from London. But whether on account of its tedious conveyance hither, or the want of experience in the committee, as yet but little acquainted with this kind of inoculation, this matter, after having been uſed with ſome ſucceſs, at length periſhed in their hands. The arrival of Dr. Woodville, Phyſician to the Hoſpital for Inoculation at London, ſoon enabled the committee to reſume their experiments.

This celebrated inoculator, who was detained at Boulogne by the formalities neceſſary for obtaining his paſſport, had inoculated ſome children in that quarter. This opportunity afforded the committee the means of procuring matter in the ſpace of twenty-four hours, as freſh as it was poſſible to obtain. More children were inoculated in the preſence of Dr. Woodville, and others ſucceſſively ſince that time.

Theſe inoculations, performed with the matter received from Boulogne, have generally manifeſted a more regular progreſs, and more determinate effects, than thoſe which preceded them; and

---

whoſe ſplendid and ſolid talents, with equal ſucceſs, have been employed in reſtoring the health of the living, and in embalming the memory of the dead. By ſome, Dr. Currie, in writing the life, may be ſuſpected of having thrown an undue luſtre on the character of the deceaſed Burns; but genius recently departed may be compared to the ſun, which never is obſerved to ſhine ſo beautifully as juſt after it has ſunk below the horizon.

the committee regard the experiments which they have made fince that time as more to be depended upon. In all the fubjects of thefe experiments, as well as in the firft, the difeafe has been of the mildeft kind; not fo much as one unfavourable accident took place. At prefent the number of inoculations performed under the infpection of the commitee amounts to one hundred and fifty.

The committee have been no lefs induftrioufly employed in putting under inoculation for the Small-pox, many of the patients who had before been inoculated with the Cow-pox, and whom they confidered as having been all, more or lefs, infected with it.

Four of thefe children were immediately inoculated on the 3d of Fructidor, three months after the infertion of the vaccine matter. About the 15th, a fecond exporiment was made upon four of them; and upon feven more on the 30th of the fame month; about two months after their firft inoculation. Laftly, on the 11th of Vendemiaire, four other children have been inoculated after the fame interval.

Of the firft four children, three have experienced not the fmalleft effect from the inoculation. The four who underwent the fecond trial, have alfo been totally unaffected wi h it. The fame has been the cafe with the other feven children. On five of thofe who were laft inoculated, and on one of the firft four, fome effects were obferved about the place of infertion; which were, that fome of them were inflamed, and that there enfued a local tumor, which terminated in fuppuration. In one of thofe five children, (named Blondeau, who had been inoculated with vaccine matter, before the arrival of Dr. Woodville,) the local tumor was attended with a degree of fever. The others were entirely free from this fymptom; and upon none of them was there any appearance of general eruption.

In order to afcertain the nature of the humour produced by this inflammation of the wounded part, the committee took fome matter from one of the fubjects of thefe experiments, and made ufe of it for inoculating two children who had never had the fmall-pox. The operation was followed by evident marks of variolous infection, fuch as is obferved in the common procefs of inoculation, accompanied with manifeft fever, and a general eruption. The committee are at this moment repeating the fame experiment upon the other four children, in whom the infertion was followed by a fmall tumor; and it fhall be renewed every time that the fame appearance occurs.

Such are the facts which the committee have obferved, fince their laft report to the public and the fubfcribers. They are far from regarding them as fufficient to eftablifh any decided conclufion on the fubject. They feel too fenfibly the importance of the queftion fubmitted to their examination, not to confider it with all the deliberation, and all the circumfpection which it requires; and their intention is ftill to continue their experiments, But there is ground for fome ftrong inductions from the facts already collected; and the committee are of opinion that they do not violate the character with which they are invefted, when they take upon them to to make the following obfervations. 1. The vaccine affection appears to them to be of a nature diftinct from that of every other kind of eruption, and particularly different from the ordinary Small-pox.

2. The vaccine affection appears to them to be of a nature the moft benign, and which hardly deferves to be called a malady. Not fo much as one accident happened to the hundred and fifty fubjects who have been inoculated.

3. This affection appears to them not to be contagious, either by the air or by contact. Children who kept company with one another during a long fpace of time, were fucceffively inoculated, and the infection manifefted itfelf in none of them before their inoculation.

4. This difeafe excites no general eruption. In all the experiments which have been made, no puftules have appeared, except at the place of infertion, and even there never more than a fingle puftule.

5. The Vaccine Inoculation is no lefs practicable than exempt from accidents, whatever be the age of the perfons on whom it is performed. Infants have been inoculated in the arms of their nurfes; others at the age of one, two, and three years, to the age of fifteen. Perfons of the age of forty, and even fifty years, have alfo been inoculated, and always with the fame fuccefs.

6. In fine, the committee have remarked a prefervative effect in the re-inoculations performed for the fmall-pox. The nineteen fubjects fubmitted to the operation, have been inoculated with frefh pus, taken every time from a variolous infant who was prefent. The committee, for the purpofe of rendering their experiments more decifive, employed in many of the fubjects very deep incifions, fuch as, according to the inoculators, neceffarily occafion a large eruption of puftules. They even proceeded fo far as to introduce, at different times, a great quantity of variolous matter into the incifions, notwithftanding which, not one of nineteen who were inoculated, had any thing like a general eruption. In fourteen, the incifions were foon obliterated, without any fymptom of complaint. In the remaining five, the inflammation can be confidered in no other light, than as the effect of local irritation, produced by the puncture of the fkin. The inflammation began the very day of the infertion. This procefs has been more rapid, and lefs regular than that of ordinary inoculation. Befides, inftances of the fame effect occur in perfons, who, after having had the fmall-pox, have again fubmitted to inoculation. In a word, if fome prefervative effect did not operate by the Vaccine Inoculation in thofe who have fubmitted to it, how fhould it happen that the variolous matter introduced into their incifions for the inoculation of the fmall-pox, fhould have excited (in fome at leaft) only a local and partial affection, whilft taken from this fource to be tranfmitted to infants not before inoculated with the Cow-pox, it has produced in the latter all the ufual fymptoms of general infection?

Thefe preliminary remarks, which, without deciding any thing on on the ſubject, the committee ſubmit to the conſideration of the learned, coincide entirely with the reſult of the experiments made at Geneva, by Dr. Odier, and of which he gives an account, in a report lately publiſhed by the prefect of that department. In ſix hundred children inoculated with vaccine matter, the mildneſs of the diſeaſe, its regular and invariable progreſs, its characteriſtic of being void of contagion, and the abſence of every complaint ſubſequent to inoculation, were conſtantly manifeſted. One very remarkable circumſtance, at the ſame time, has afforded an opportunity of evincing its preſervative operation. An epidemic ſmall-pox, of a very malignant kind, having appeared at Geneva, where upwards of a hundred and fifty children were victims to it, and where ſeventy-ſix more have died within the laſt month, it was obſerved that the children who had been inoculated with the Cow-pox, were totally unaffected by the contagion, except about ſeven or eight only, who had contracted the infection before their inoculation, and in whom the ſmall-pox had made their appearance on the fourth or fifth day after the inoculation with the Cow-pox, which in conſequence of that accident became uſeleſs.

In the Name of the Committee for Vaccine Inoculation,

THOURET, Director of the Medical School.

---

### COW-POX.

The practice of Vaccine Inoculation, though of recent origin, has already forced itſelf upon the notice of the firſt profeſsional characters in this and the neighbouring kingdoms. Its advantages are too numerous to be detailed here; but it may be proper to obſerve, that the vaccine diſeaſe requires no preparative medicine, no change of regimen, no confinement, and is ſeldom attended with much indiſpoſition.

We admit that, comparatively, few have periſhed by Small-pox Inoculation; but it is conſiſtent with our experience, that numbers have been ſo dangerouſly ill, as to excite ſerious alarms for the iſſue of the complaint. Under the circumſtances of Vaccine Inoculation, no ſymptom of danger has ever been exhibited. The Small-pox is a diſeaſe of contagion. The Cow-pox cannot be communicated but by actual contact or inciſion; which circumſtance enables the inoculator to ſingle out any individual for the operation, without ſubjecting any other member of the family to the ſmalleſt riſk.

That the Cow-pox is a ſubſtitute for the Small-pox is now ſufficiently certain. The experiments which have been inſtituted to put the queſtion beyond all doubt, are numerous, concluſive, and ſatisfactory.

Impreſſed with a conviction of the truth of thoſe facts, and animated by no other motive than a ſenſe of duty, and a ſincere deſire to reſcue the lower orders of ſociety from the ravages of a contagion, which to them is ſo peculiarly diſtreſſing and fatal, the underſigned Surgeons are deſirous to introduce a diſeaſe infinitely more mild, and very certainly ſafe: They are therefore ſolicitous to recommend to the inhabitants of Bradford and its vicinity, the the practice of Vaccine Inoculation; and they hereby inform the public, that they will attend at their own houfes every Tuefday and Friday morning, to Inoculate, gratis, the children of the poor; and Dr. Moffman, fhould any indifpofition occur, or other circumftance requiring extraordinary attention, will give his joint assistance in carrying every perfon fo inoculated, fafely through the difeafe.

GEORGE MOSSMAN, M. D.

THOMAS JONES, Surgeon.

W. MAUD, } Surgeons.

THOMAS LISTER, }

*Bradford, Dec.* 4, 1800.

---

The following fignatures are added to the teftimonial in favour of Vaccine Inoculation.

| | |
|---|---|
| Nathaniel Hulme, M. D. | James Gilder |
| Gilbert Blane, M. D. | John Griffiths |
| William Blackburne, M. D. | James Higgins |
| Sir J. M'Namara Hayes, Bart. M. D. | Lewis Leefe |
| | Edward Leefe |
| Andrew Thynne, M. D. | William Lynn |
| Edward Fryer, M. D. | John Mackinder |
| Sayre Walker, M. D. | Jonas Malden |
| Michael Underwood, M. D. | Jofeph Millington |
| Thomas Garnett, M. D. | William Morris |
| John Gibfon, M. D. | John Pearfon |
| G. M Burrowes | Thomas Rolph |
| David Dundas | John Rufh |
| Thomas Farquhar | Stephen Woolriche. |
| Henry Fearon | |

---

Dr. BRADLEY will commence his Spring Courfe of Lectures on the Theory and Practice of Medicine, on Wednefday, Jan. 21, 1801, at fix in the afternoon.

---

Dr. BATTY will begin his Winter Courfe of Lectures on the Theory and Practice of Midwifery, and on the Difeafes of Women and Children, on Monday, January 26, 1801, at half paft ten o'clock in the morning.—Further particulars may be known by applying to Dr. Batty, at his houfe, No. 6, Great Marlborough Street.

---

Mr. PEARSON will commence his Spring Courfe of Lectures on the Principles and Practice of Surgery, on Monday, Jan. 26, 1801, at feven o'clock in the evening, at his houfe in Golden Square.

---

Mr. H. LEIGH THOMAS propofes delivering a Courfe of Lectures on Practical Surgery, in which the different operations will be performed, and the medical treatment fully explained.

The Courfe will commence on Monday, January 26, 1801, at ten minutes after eight o'clock in the evening, at his houfe in Leicester Square, where further particulars may be known, or at the Anatomical Theatre in Great Windmill Street.

¶. We have inserted the above at the reqeust of Mr. Thomas, and we conceive, that a course of Lectures which will embrace a full consideration of the medical practice requisite in Surgery, (a most essential part, and which has not been sufficiently detailed hitherto) will prove of the utmost value and assistance to the student. The medical world is sufficiently acquainted with the anatomical and professional talents of Mr. Thomas, as well as his opportunities for illustrating his subjects from the collection of preparations of the late celebrated Mr. Cruikshank, and which he has so considerably enlarged.

---

Mr. Wilson will commence his Spring Course of Lectures on Anatomy, Physiology, Pathology, and Surgery, on Jan. 19, 1801, at the Theatre of Anatomy in Great Windmill Street.

---

Mr. Chevalier's Spring Course of Lectures on the Principles and Practice of Surgery, will commence at his house, No. 20, in South Audley Street, Grosvenor Square, on Monday, Jan. 26, 1801, at seven o'clock in the evening.

---

Mr. Stancliffe, of Cambridge, Lecturer in Chemistry to the Middlesex Hospital, proposes to deliver the same Course to an audience in Birmingham, in the course of the ensuing month.

---

Dr. T. A. Murray, of Greville Street, Hatton Garden, will shortly publish "Remarks on the Situation of the Poor in the Metropolis, as contributing to the progress of contagious diseases; with a plan for the establishment of houses of recovery for persons infected by fever."

---

We learn that the second edition of Mr. Parkinson's Chemical Pocket-Book, printed on a fine wove paper, and augmented by the addition of the latest discoveries, is expected to be published in the beginning of January.

